# An improved internal mammary irradiation technique in radiation treatment of locally advanced breast cancers

**DOI:** 10.1120/jacmp.v6i1.2079

**Published:** 2005-03-17

**Authors:** Jian‐Yue Jin, Eric E. Klein, Feng‐Ming Kong, Zuofeng Li

**Affiliations:** ^1^ Department of Radiation Oncology, Siteman Cancer Center Washington University Medical Center St. Louis Missouri 63110 U.S.A.; ^2^ Present address: Henry Ford Hospital, 2799 W. Grand Blvd. Department of Radiation Oncology Detroit MI 48202 U.S.A.; ^3^ Present address: The Department of Radiation Oncology University of Michigan Medical Center Ann Arbor MI 48109 U.S.A.

**Keywords:** internal mammary nodes, breast radiotherapy, radiation technique, field matching, lung and heart toxicities, DVH, SMLC

## Abstract

The purpose of the present study was to compare a new internal mammary irradiation technique with traditional techniques for locally advanced breast cancers in terms of sparing ipsilateral lung and heart and reducing the “cold” and “hot spots” in breast tissue. The new technique uses wide tangential fields for the first eight fractions of treatment. A medial internal mammary field (IMF) of electrons matched with narrowed tangential fields is used for the remaining fractions. Intensity‐modulated radiation therapy (IMRT) by means of segmented multileaf collimation (SMLC) is used in the narrowed tangential fields to improve the match between the electron and the photon fields. Treatment planning was performed to compare this technique to a wide‐tangential‐only technique and to a traditional oblique IMF technique for three patients with differing habitus. Film dosimetry was performed in a solid water phantom to confirm the planning results. For all three patients, the mean doses of the ipsilateral lung and the heart were significantly reduced with the new technique. The lung and the heart volumes were remarkably reduced at low‐dose levels (≤12GY) compared to the traditional IMF technique, and significantly reduced at all dose levels compared to the wide tangential technique. The new technique also reduced the “cold” and “hot spots” along the match plane between the IMF and the tangential fields compared to the traditional IMF technique. In conclusion, the new IMF technique shows dosimetric improvement compared to the traditional IMF technique in terms of the critical organ sparing and target dose uniformity.

PACS number: 87.53.Tf

## I. INTRODUCTION

Randomized trials have demonstrated the benefits of irradiation to the regional lymph nodes for locally advanced breast cancers.^(^ ^1^ ^–^ ^3^ ^)^ However, irradiation to the internal mammary nodes (IMN) remains controversial among radiation oncologists due to the concern of potential lung and heart toxicities. Several techniques have been developed to address this issue. These include a partially wide tangential technique,^(4)^ an enface internal mammary field (IMF) technique,^(5)^ an oblique IMF technique,^(6)^ ^,^ ^(7)^ a multisegment abutting electron field technique,^(8)^ and an electron arc technique.^(9)^ These techniques have been compared clinically and/or dosimetrically by many investigators.^(^ ^9^ ^–^ ^14^ ^)^

In our institution, we had frequently used the oblique IMF technique to treat the IMN and chest wall for patients indicating regional lymph node irradiation. In many cases, we also used this technique to treat the intact breast for patients with large central lung distance; in such cases, the IMF was essentially a medial breast portal.^(6)^ A combination of electron and photon beams was used for the internal mammary field with the photon:electron ratio of 8:18. The photon beam was matched with narrowed tangential fields with the exact same gantry angle, while the electron beam was angled 5° medially compared with the tangential fields. The photon:electron ratio was determined historically, mainly to balance the skin dose, the “hot/cold spots” along the matching plane, with the dose to the lung and heart.^(6)^ This technique provided reasonable dose coverage to the target with a reduction of percentage volumes of the lung and heart receiving a high dose (>20Gy).^(6)^ However, there are two caveats in this technique: (1) a remarkably large volume of lung and heart received low dosage (<10Gy); and (2) a mismatch of the penumbra between the electron beam of the IMF and the photon beam of the tangential fields introduced a “cold spot” within the IMF irradiated volume and a “hot spot” inside the tangential field irradiated volume.

The purpose of this study is to improve our current technique with the following modifications: (1) wide tangential fields with increased obliquity replacing the IMF photon beam that abuts with the narrow tangential fields; and (2) simple intensity‐modulated radiation therapy (IMRT) by means of segmented multileaf collimation (SMLC) is used for the narrow tangential fields to improve the match between the IMF electron and tangential photon beams.

## II. MATERIALS AND METHODS

CT images of three left breast cancer patients with different geometry and sizes were used in this study. Targets and organs at risk were delineated by physicians. The ipsilateral breast was contoured based on visible breast tissues and clinically marked borders on CT images. The ipsilateral IMN from the first to fifth intercostal spaces were contoured according to the lymph nodes atlas outlined by Matrinez‐Mage.^(15)^ The left lung (ipsilateral lung) was contoured using a density‐seeking tool, with exclusion of hilum and tracheas. The heart was contoured using all the soft tissue shadow starting at one slice below the inferior cut of the right pulmonary artery crossing the midline.

**Figure 1:**
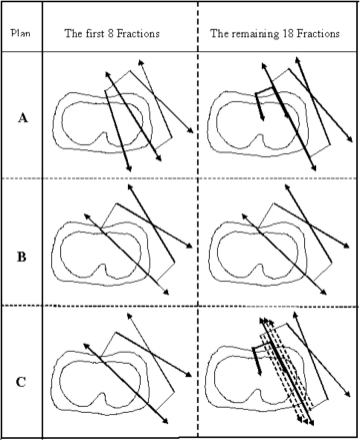
Schematic diagrams of each treatment technique. Plan A: First 8 fractions, IMF photon matching with narrow tangential fields; remaining 18 fractions, IMF electron matching with narrow tangential fields. Plan B: First 8 fractions, wide tangential fields; remaining 18 fractions, wide tangential fields. Plan C: First 8 fractions, wide tangential fields; remaining 18 fractions, IMF electron matching with SMLC modulated narrow tangential fields.

Treatment planning was performed using a 3D treatment‐planning system (FOCUS v3.1, Computerized Medical Systems, St. Louis, MO). Two dimensional compensation (Ellis type) filters were used for all tangential fields. For consistency, no inhomogeneity correction was used. The dose contributions from a supraclavicular field and a posterior‐anterior axilla field were not included. Three plans (A, B, and C) were performed for each patient for comparison with a dose of 46.8 Gy in 26 fractions to the same prescription point (Fig. 1).

Plan A used the oblique IMF technique currently practiced in our institution, in which an IMF abuts with two parallel opposing narrow tangential fields. A combination of electron and photon beams with the photon:electron ratio of 8:18 was given to the IMF. A half‐beam technique was used in the transverse plane (X‐jaws) for both the IMF photon and the tangential fields so that there was a proper match between the IMF and medial tangential photon beams. The isocenter was at the “midbridge” of the narrow tangential fields along the midbreast plane. The prescription point was 2.5 cm anterior‐lateral to the isocenter for the tangential fields, and at the depth of the chest wall in the middle of the field for the IMF. The energy of all photon beams was 6 MV. The energy of the electron beam varied from 9 MeV to 16 MeV according to the thickness of the chest wall for different patients. Patient 1 used an electron energy of 16 MeV and a prescription depth at 90% dose line for the IMF electron beam. Patient 2 used an electron energy of 12 MeV prescribed at the depth of maximum dose for the IMF electron beam. Patient 3, a postmastectomy patient, used an electron energy of 9 MeV and a prescription at the depth of maximum dose for the electron beam. For this technique the IMF electron field was matched with the tangential fields on the skin surface. The gantry was angled 5° medially compared with the tangential fields to best compensate for the bulge of electron isodose curve to the tangential fields.

Plan B used a simple, wide tangential technique with the same isocenter as plan A (Fig. 1). The closed medial transverse (X) jaw was opened 3 cm to 5 cm to include the IMN in the tangential fields. The gantry angle of the medial tangential field was pitched obliquely compared to the corresponding field in plan A. The medial and lateral tangential fields were pitched several degrees, so that the projections of their medial/posterior edges were congruent. The prescription point (weight point) was also 2.5 cm anterior/lateral to the isocenter.

Plan C used the proposed new technique. For the first 8 fractions of treatment, wide tangential beams similar to those in plan B were used. The remaining 18 fractions used an IMF electron beam matched with two narrowed tangential fields, similar to those in plan A. However, IMRT by means of SMLC was used in the narrowed tangential fields to blur the sharp photon penumbra. Each SMLC field was composed of four segments (subfields). For the medial tangential field, the medial edge of each subfield was opened 0 mm, 3 mm, 6 mm, and 9 mm, respectively, compared to the initial medial field. For the lateral field, the posterior edge of each subfield was closed 0 mm, 3 mm, 6 mm, and 9 mm, respectively, compared to the initial lateral field. Each subfield was assigned with a dose weight one‐quarter of the original dose for the tangential fields. The medial tangential field was relatively opened to compensate for the “cold spot” on the chest wall because the depth of the “cold spot” for the medial tangential beam was much shorter than its depth for the lateral tangential beam. On the other hand, the lateral tangential field was relatively closed to reduce the “hot spot” on the lateral posterior aspect of tissue, which in many times was actually not breast tissue. However, this arrangement of field‐intensity modulation was far from optimized. For example, closing the lateral subfields could potentially reduce the coverage of the target on the lateral posterior aspect, if the posterior margin of the lateral field to the target was tight.

After optimization and evaluation of the treatment plans, the beam parameters (beam energies, gantry angles, collimator setting, field shapes, etc.) for plans A and C were recalculated for a solid water phantom, and corresponding phantom plans were generated. The MLC shapes were exported to the “Shaper” program (Varian Medical Systems, Palo Alto, CA). The four MLC subfields were grouped together to form a composite SMLC field. The SMLC fields were then exported to a Varian EX‐21 linac (Varian Medical Systems, Palo Alto, CA) and to deliver dose to the solid water phantom. Since the leaf‐traveling distance between each subfield was very short, the time between each segment during the SMLC delivery was minimal. The total delivery time for the composite SMLC field was approximately the same as for a standard tangential field.

Films (silver bromide, XV‐2, Kodak, Rochester, NY) were placed on the axial plane for film dosimetry verification during dose delivery to the solid water phantom. Monitor units were scaled down so that the maximum dose was in the linear region of the XV film. Known doses were also delivered to a series of films to generate a dose‐to‐density calibration curve. The films were developed and scanned using a densitometer (Dynascan, CMS, St. Louis, MO).

## III. RESULTS

### A. Treatment‐planning data

#### A.1 General treatment‐planning data

Table 1 summarizes the treatment‐planning results using different techniques for all three patients. Although the three patients have very different sizes and geometry, the treatment‐planning results for different techniques show similar trends for all three patients. Plan C has the lowest mean doses for the ipsilateral lung and the heart for all patients, while plan B has the highest mean doses for these critical organs. Compared to plan A, plan C also has lower maximum doses and a higher minimum dose in the vicinity of the match plane for all patients. The dose coverage to the breast and to the IMN is comparable among all plans but varies for different patients.

**Table 1:** Summary of treatment‐planning parameters and results for three different plans for three patients

	Plan	Electron energy (MeV)	Mean lung dose (Gy)	Mean heart dose (Gy)	Hot spot^a^ (Gy)	Cold spot^b^ (Gy)	Breast coverage^c^	IMN coverage^c^
patient 1	A	16	15.7	12.5	60.1	40	95%	84%
	B	N/A	22.0	15.9	51.8	N/A	88%	93%
	C	16	13.4	10.9	55.3	45	94%	86%
patient 2	A	12	14.4	11.2	54.5	39	91%	96%
	B	N/A	18.3	11.6	53.3	N/A	95%	85%
	C	12	11.7	7.4	53.1	45	93%	93%
patient 3	A	9	17.8	9.3	62.5	40	90%	89%
	B	N/A	28.7	15.7	56.6	N/A	95%	100%
	C	9	16.7	7.2	56.0	45	93%	93%

^a^ Hot spot dose is the globe maximal dose.

^b^ Cold spot dose is the isodose line dose that encompasses the breast tissue near the match line.

^c^ Breast and internal mammary node (IMN) coverage is defined as the percentage volume covered by the prescription dose.

#### A.2 Ipsilateral lung dose‐volume histogram

**Figure 2:**
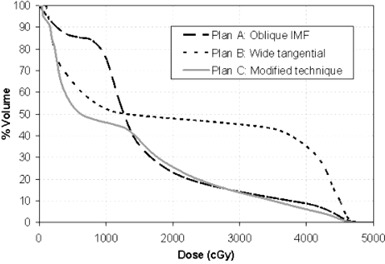
Dose‐volume histograms of the ipsilateral lung for three different techniques

Figure 2 shows the comparison of dose‐volume histograms (DVH) of the ipsilateral lung among three plans for patient 1. The wide tangential technique (plan B) shows minimal sparing of the ipsilateral lung. The percentage lung volume at 40 Gy dose levels reaches approximately 40%. Such a high volume/dose level is not acceptable due to possible lung complications. The oblique lique IMF technique (plan A) shows a large reduction of the ipsilateral lung volume for dose >20Gy. However, the volume of lung at low‐dose regions (i.e., <12Gy) increases significantly. The modified technique (plan C) shows significant reductions of lung volume at all dose levels compared to plan B. It also shows a remarkable reduction of lung volume at a low‐dose region (<12Gy) and a slight reduction of volume at a higher‐dose region (<30Gy) compared to plan A. There is a slight increase of volume at the dose level of about 20 Gy, due to the blurring of photon penumbra by using SMLC. Similar lung DVH behaviors were found for the other two patients.

### A.3 Heart dose‐volume histogram

**Figure 3:**
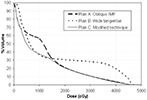
Dose‐volume histograms of the heart for three different techniques

Figure 3 shows the comparison of DVH for the heart among the three plans for patient 1. It exhibits a similar DVH behavior as was observed for the ipsilateral lung. Again, plan B shows a substantially higher volume of heart receiving doses in the range of 20 Gy to 40 Gy. Plan A shows a remarkable reduction of the heart volume at higher doses, but a significant increase of the volume in the low‐dose region. Plan C apparently shows the best heart sparing with reduction of the heart volume at all dose levels compared to both plan A and plan B. Similar heart DVH features are found for the other two patients.

### A.4 Target dose‐volume histograms

**Figure 4:**
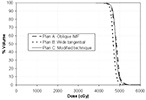
Dose‐volume histograms of the breast tissue for three different techniques

Figure 4 shows the comparisons for DVHs of the breast tissue among three different plans for patient 1. The coverage of the breast tissue is comparable for all three plans. Plan B has the least mean dose but the best dose homogeneity for breast tissue. This is because the “hot spots” are not inside the contoured breast tissue for the wide tangential technique.

**Figure 5:**
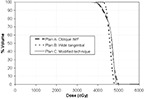
Dose‐volume histograms of the internal mammary nodes for three different techniques

Figure 5 shows the comparisons of DVHs of the IMN among three plans for the same patient. The coverage of the IMN is reasonable for all plans. However, some regions of the IMN receive less than 40 Gy (90% of prescription dose) for plans A and C. This is because the IMN/chest wall has different depths in different intercostals levels. The chest wall is usually thicker in the superior levels than in the inferior levels. The electron energy may not be high enough for the thicker chest wall in the superior levels. For patient 2, using 12 MeV electron energy, the IMN coverage was found to be better for plans A and C than plan B (see Table 1).

### A.5 Isodose distribution

**Figure 6:**
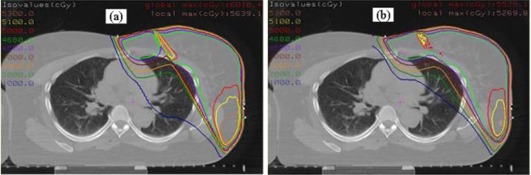
Two‐dimensional isodose distributions in an axial cut from treatment planning for (a) plan A and (b) plan C

Figures 6(a) and 6(b) show isodose distributions of plan A and plan C for patient 1, respectively. The match between the electron and the photon fields is significantly improved in plan C compared with plan A. A “hot spot” with globe maximum of 60.1 Gy and the local maximum of 56.4 Gy is observed in the vicinity of the match plane for plan A, compared to the globe maximum of 55.2 Gy and the local maximum of 52.4 Gy for plan C, in which the maximum dose is not in the vicinity of the matching plane. In addition, plan A shows a remarkable “cold spot” at the side of the electron field beside the match plane, while plan C shows the prescription dose line encompassing the breast tissue nicely near the chest wall. The isodose distributions for the other two patients exhibit similar features.

### B. Film dosimetry data

**Figure 7:**
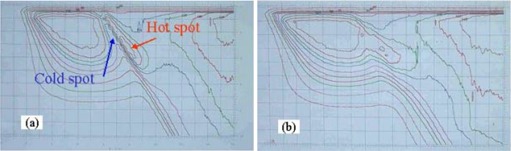
Two‐dimensional isodose distributions in an axial cut from film dosimetry measurement for (a) plan A and (b) plan C

The treatment‐planning parameters of plans A and C for patient 1 were recalculated for a solid water phantom, and corresponding phantom plans were generated. Doses delivered to the axial planes of the phantom were analyzed. To emphasize the matching behavior of the electron field and photon field, isolated measurements were performed with dose delivery only corresponding to the fractions of the IMF electron matched with the tangential photons (omitting the fractions of photon‐photon match). Figures 7(a) and 7(b) show these isodose distributions corresponding to plan A and plan C, respectively. They demonstrate that without using the SMLC (plan A), there is a severe “cold spot” at the side of electron field and a “hot spot” at the side of photon field along the match plane. By using the SMLC (plan C), the match between the electron and the photon fields is remarkably improved. This confirms that the improvement of match demonstrated by the treatment planning can be achieved in the real‐time dose delivery.

The isodose distributions corresponding to an entire phase of dose delivery were also measured. They were found to be in good agreement with the isodose distributions in the phantom plans. These suggest that the modeling of the electron penumbra data in our treatment‐planning system is acceptable.

**Figure 8:**
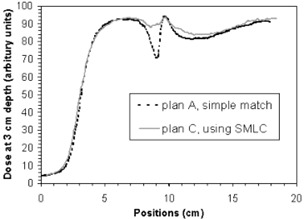
Comparison of measured dose profiles for plan A and plan C in an axial cut at the depth of 3 cm

**Figure 9:**
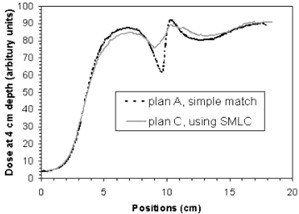
Comparison of measured dose profiles for plan A and plan C in an axial cut at the depth of 4 cm

Figure 8 and Fig. 9 show the comparison of measured dose profiles at depths of 3 cm and 4 cm, respectively, for plans A and C corresponding to dose delivery of the last 18 fractions (IMF electron matched with narrow tangential fields). Positions at the *x*‐axis represent points from right to left in the central axis cut in the irradiated area. The position at x~9.5 cm corresponds to a point at match line. They show that using SMLC in the match between the photon and the electron beams significantly improves the dose homogeneity, and especially reduces “cold spot” near the match line at the prescription depth (3 cm to 4 cm for 16 MeV electron), compared to the conventional match method.

## IV. DISCUSSION

Our modified technique achieves better sparing of the lung and the heart by using wide tangential fields with a relatively large oblique gantry angle instead of an IMF photon matched with narrow tangential fields. In addition, the target dose coverage and homogeneity were significantly improved by using the SMLC technique in the match between the electron and photon fields.

Electron and photon beams have different penumbra and dosimetric behaviors. Simply matching electron with photon beam causes “cold” and “hot spots” along the match plane. Li et al. had used intensity‐modulated inverse treatment planning to match the photon fields with the electron field.^(16)^ Their results show that a perfect match at a defined depth could be achieved with this method. They also demonstrated that this method was more tolerable for setup errors. This study uses concepts similar to Li et al.'s work to match the electron field with the photon fields, except a rather simple or primitive method to generate the intensity‐modulated photon fields. The results of this study are consistent with the previous work. Because no inverse treatment planning was involved, the technique proposed in this study can be used in clinics without inverse treatment‐planning systems.

The breasts and the chest wall are targets that move with patient respiration during the treatment. It was believed that the organ motion could have an effect of intensity modulation within the abutting fields and potentially wash out the “hot” and “cold spots.” The results of our study partially support this thought. However, this is a random modulation and cannot be controlled. With a controlled intensity modulation superimposed with a random modulation, the organ motion effect could improve the dose uniformity along the matching plane for the technique proposed in this study.

This paper does not include the partially wide tangential technique for comparison because the target definition for this technique is different.^(11)^ The partially wide tangential technique usually does not irradiate internal mammary nodes below the third intercostal level, and it could partially block breast tissue and axilla nodes below this level. Whether to irradiate the IMN and the axilla nodes at lower level is a clinic judgment; however, for the upper part of the target, in terms of irradiation technique, the partially wide tangential technique should have the same dosimetric behavior as the wide tangential technique.

It must be pointed out that the medial wide tangential field with a large oblique angle could partially irradiate some of the contralateral breast tissue. Therefore, a reasonable gantry angle has to be chosen to balance between sparing of the heart and the lung and sparing the contralateral breast.

## V. CONCLUSION

We have developed a new technique for the treatment of the internal mammary nodes in breast radiotherapy. Wide tangential fields with a larger oblique angle replace an IMF photon beam matched with the narrow tangential fields to reduce the irradiated lung and heart volumes. The SMLC technique is used in the narrow tangential fields to better match the IMF electron beam. Treatment‐planning results demonstrate that the new technique reduces the dose/volume of the ipsilateral lung and heart and improves the target coverage (by reducing the cold spot) and dose homogeneity. Film dosimetry also confirms that this technique significantly improves the match between electron and photon fields.

## Supporting information

Supplementary Material
