# Supplementary material for: An improved internal mammary irradiation technique in radiation treatment of locally advanced breast cancers
Source: J Appl Clin Med Phys. 2005 Mar 17;6(1):84–93. doi: 10.1120/jacmp.v6i1.2079 (PMC5723509; doi:10.1120/jacmp.v6i1.2079)
Supplement: Supplementary file 1 — Supplementary Material [file ACM2-6-084-s001.doc]

**An Improved Internal Mammary Irradiation Technique in Radiation Treatment of Locally Advanced Breast Cancers**

Jian-Yue Jin^a^, Ph.D., Eric E Klein, MS, Feng-Ming Kong^b^, MD, Zuofeng Li, D.Sc.

Department of Radiation Oncology, Siteman Cancer Center, Washington University Medical Center, St Louis, MO 63110

1. Currently in Henry Ford Hospital, 2799 W. Grand BLVD, Department of Radiation Oncology, Detroit, Michigan, 48202. [Jjin1@hfhs.org](mailto:Jjin1@hfhs.org)
2. Currently in The Department of Radiation Oncology, University of Michigan Medical Center, Ann Arbor, Michigan 48109
